# The Progress of Cremation in America

**Published:** 1896-05-23

**Authors:** 


### Some Aspects of Sanitation.

### THE PROGRESS OF CREMATION IN AMERICA.

Americans are inclined to take strong views on most subjects, and the question of cremation amongst our Transatlantic cousins forms no exception to the rule. Moreover, in regard to this method of disposal of the dead they not only take strong views, but they make strong statements, and they have begun to act with considerable energy.

In matters sanitary England has long been accustomed to take and keep the lead, but with regard to that most important sanitary matter, namely, the disposal of the dead, although some time back we made a brave attempt at popularising cremation, the progress has been so slow that we may almost be said to have been left standing by the more enterprising Americans. Coercion of any kind is unpopular in England, and nothing probably would be received with greater disfavour than a reform so radical in its nature as compulsory cremation of the dead. But there can be no reasonable objection to the disposal by workhouse authorities of the dead inmates by a means which has so much to recommend it from a sanitary standpoint. It is with considerable satisfaction that we learn that this question has been seriously taken up by the authorities of a great centre of population in America. The step which has thus been initiated is one which will doubtedly be followed by other townships, and it is with hopeful anticipation that we read that in Philadelphia the indigent dead are no longer consigned to a potter's field, and the groaning earth is thus relieved of some portion of its burden of purification.

If, say these advocates of cremation, you commit a body to the earth, you must presuppose its final resolution into its elementary chemical units. The process may, indeed, be a slow one, but the further you prolong the destructive action of nature by confinement in brick graves and resistant coffins, the more opportunities you allow for the dissemination of foul gases, poisonous leucomains, and other foul products of organic decay. Sentiment becomes crass folly when it rejoices in decadence which is extended over centuries, and weeps when the purging element of fire completes the destruction in a few minutes. Sentiment as well as custom and usage must be allowed its due weight in the ordering of mortal affairs, and hence, say our practical brethren across the water, sentiment is the only bar to cremation. Let us cremate those with whom sentiment is unconcerned; let us, in fact, burn our unwept-for and pauper dead; let us also, they continue, indulge the fancies of our fellow citizens, and permit them to dispose of their own dead in whatsoever manner pleases them best, unfettered by personal or public prejudice, provided that in so doing they cause no nuisance inimical to the health of others. And so in America we learn of facilities for the incineration of the dead established in California, Los Angeles, Cypress Lawn, San Mateo county, New York, Buffalo, Troy, and many other cities. And in Philadelphia, as we have above mentioned, the paupers are one and all cremated, whether death is due to contagious or other disease.

In these days prophylaxis against disease is regarded as the most rational and most effective force in medicine, and hence when we spend millions annually in maintaining the purity of the water supplies, and are very jealous of the slightest befoulment of the air by emanations from factory chimneys or from unhealthy manufactories, it seems somewhat paradoxical to leave earth, the third element of this important trio, to the undisturbed enjoyment of all the corruption and filth that public bodies or private individuals may think fit to pour into its recipient maw.

There are practically only two solid arguments against cremation, the first being of no very substantial order, namely, the sentimental objection of individuals, and the second the question of cost. Time will dispose of the former; this will entail increased demand for facilities, with the consequent disappearance of the second objection.

It is true that argument may be drawn from the economics of nature. But this theoretical and most problematical disadvantage cannot be fairly weighed against the material gain—a real sanitary reform, to say nothing of the gain of space for building, cultivation, or recreation. The argument to which we refer is the destruction of fixed nitrogen which occurs in the combustion of any animal unit. Such a procedure as cremation detracts from the available store of fixed nitrogen, to which animals and plants owe their existence. The total sum of organic life must be thereby ultimately diminished, and the fertility of the soil similarly decreased. Every time the stoker at Woking kindles his furnace for the destruction of a human body he is guilty of drawing upon the capital of nitrogen, upon which depend the total number of living beings that are or are to come.

Those who by reason of sentimental objections inveigh against the practice of cremation, are advised by one American enthusiast—not indeed for their own personal advantage but for the benefit of the community at large—to be present at the exhumation of a body that has been in mother earth for a year or two; he avows that they would never be buried themselves, or recommend to their friends or relations a similar means of corporeal exit from this world. One experience would dispel all sentiment, the mind would for ever afterwards revolt against the usage. The eye cannot behold or the mind imagine a more repulsive, shocking, or hideous sight. The furnace and the stoker may not be pleasant to behold, but the grave is a grim charnel house, where creeping worms, and seething masses of bacteria enjoy a loathsome feast.

The Cremation Society of England, under the auspices of Sir Spencer Wells and Sir Henry Thompson, has since the year 1878 developed with steady but unostentatious progress, avoiding friction where possible, and showing a respect and tolerance of the prejudice and sentiments of others, conduct which in the immediate future must reap a harvest of success no less plentiful than that which has been garnered on the other side of the Atlantic.

